# Supplementary material for: A Universal Model of Commuting Networks
Source: PLoS One. 2012 Oct 1;7(10):e45985. doi: 10.1371/journal.pone.0045985 (PMC3462197; doi:10.1371/journal.pone.0045985)
Supplement: Supporting Information S1 — (ZIP) [file pone.0045985.s001.zip › SupportingInformation.pdf]

Supporting Information: A Universal Model of Commuting Networks

Maxime Lenormand[1,*], Sylvie Huet[2], Floriana Gargiulo[3], Guillaume Deffuant[4]
1 LISC, Irstea, Clermont-Ferrand, France
2 LISC, Irstea, Clermont-Ferrand, France
3 CAMS, CNRS, Paris, France
4 LISC, Irstea, Clermont-Ferrand, France
∗ **E-mail: maxime.lenormand@irstea.fr**

Data presentation

1.1 Datasets

Commuting data are usually provided by statistical offices in the form of origin-destination tables. We analyzed 80 case studies from 7 differents datasets and 4 different country (described in Table 1). In these appendices we called outside (Out.) the m units surrounding the area.

1.2 Distance

The distances between units are Euclidean, computed using the Lambert coordinates or the latitude/longitude of the centroid of the units.

1.3 Case studies

We define two types of case studies: from administrative regions and from aggregation of small administrative units around a randomly chosen point. Each case study is composed of a region and an outside (the units surrounding the region at a reasonable distance).

To build a case study from an admistrative region, we select an administrative region (For example the Auvergne region represented by the dark grey region in Figure 1a) and to build the outside we select all the units surrounding the region at a reasonable distance (For the Auvergne region example, the outside is represented by the light grey region in Figure 1a).

To build a case study by aggregation of units, firstly, we define the number of desired units and we draw at random a latitude and a longitude (For example the point represented in Figure 1b). In a second time we gradually increase the area of a square with as center the starting point until the desired number of units is obtained (Figure 1c). To build the outside we select all the units surrounding the defined set of units at a reasonable distance or all the remaining units in the country (it depends of the number of units).

The case studies with an identifier with a 0, for example $FRc0$, are complete network of the country without outside. Indeed, we have no data for the surrounding countries. When we consider a region in the country we can determine the outside as the units surrounding the region. When we consider as a region the whole country we can't determine an outside, it is the case for $FRc0$ (all the Cantons of France), $Frd0$ (all the Départements of France), $Itp0$ (all the Provincias of Italy) and $USA0$ (all the counties of USA).

1.4 Source

The 3 French datasets are measured for the 1999 French Census by the French Statistical Institute, $INSEE$. They were kindly made available by the Maurice Halbwachs Center.

The 2 Italian datasets are measured for the 2001 Italian Census by the National Institute for Statistics, $ISTAT$.

Table 1. Presentation of the datasets.
* Data are available online at `http://www.czso.cz/eng/redakce.nsf/i/home`
** Data are available online at `http://www.census.gov/geo/www/gazetteer/places2k.html`

**Dataset**	**Country**	**Case Study**	**Distance**	**Region**	**Scale**	**Year**	**Source**
1	Czech Republic	CZ	Latitude Longitude	Administrative	Municipality	2001	*
2	France	FR1 - FR34	Lambert	Administrative	Municipality	1999	INSEE
3	France	FRc0 - FR14	Latitude Longitude	Arbitrary aggregation	Canton	1999	INSEE
4	France	FRd0	Latitude Longitude	Administrative	Département	1999	INSEE
5	Italy	IT1 - IT10	Latitude Longitude	Arbitrary aggregation	Municipality	2001	ISTAT
6	Italy	ITp0 - ITp4	Latitude Longitude	Arbitrary aggregation	Provincia	2001	ISTAT
7	USA	USA0 - USA14	Latitude Longitude	Arbitrary aggregation	County	2000	**

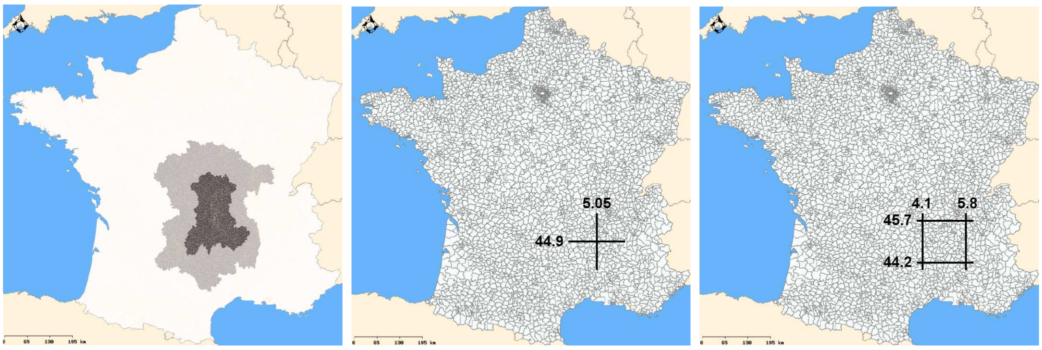

(a) (b) (c)

Figure 1. Maps to illustrate the build process regions. (a) Administrative; (b) starting point of aggregation and (c) limits of aggregated units.
Base maps source:
Cemagref - DTM - Dveloppement Informatique Systme d'Information et Base de Donnes : F.Bray & A.Torre
IGN (Gofla , 2007)

Results with standard indicators of error

We computed the results with standard indicators of error.

- The Normalized Mean Absolute Error:

$$NMAE(T,\tilde{T}) = \frac{\sum_{i=1}^{n}\sum_{j=1}^{n}|T_{ij} - \tilde{T}_{ij}|}{\sum_{i=1}^{n}\sum_{j=1}^{n}T_{ij}} \quad (1)$$

- Normalized Root Mean Square Error:

$$NRMSE(T, \tilde{T}) = \frac{\sqrt{\sum_{i=1}^{n} \sum_{j=1}^{n} (T_{ij} - \tilde{T}_{ij})^2}}{\sum_{i=1}^{n} \sum_{j=1}^{n} T_{ij}} \tag{2}$$

Examples of detailed comparison between the radiation model and our model

Table 2. Description of the case studies

Case study	Number of units (area)	Number of units (outside)	Surface (km^2)	Average unit surface (km^2)	Standard deviation unit surface (km^2)	Observed number of commuters (Area)	Estimated number of commuters (Area)
CZ	43	630	35369	822.54	703.23	6585	6847
FR1	1310	3463	26013	19.86	12.49	261822	262452
FR2	1269	1447	27208	21.44	16.14	608587	613363
FR3	419	2809	5762	13.75	8.46	90456	76829
FR4	903	3081	8280	9.17	9.55	409661	402565
FR5	2296	2835	41309	17.99	21.30	679639	657095
FR6	261	3124	5175	19.83	10.46	52921	48681
FR7	185	1859	5167	27.93	18.71	9474	8981
FR8	1464	2467	25810	17.63	12.94	333045	333540
FR9	1842	4718	39151	21.25	14.76	514461	529535
FR10	3020	3845	45348	15.02	15.74	502326	494946
FR11	747	3169	16942	22.68	14.15	118508	117217
FR12	1786	3317	16202	9.07	7.46	239931	236314
FR13	1420	3536	12317	8.67	5.64	396800	402128
FR14	433	3914	6211	14.34	12.41	30175	28729
FR15	515	3808	5874	11.41	9.54	76519	72896
FR16	2339	3067	23547	10.07	7.51	505807	507812
FR17	260	1814	5565	21.40	13.15	17310	17071
FR18	1545	3046	27367	17.71	15.78	354824	354566
FR19	1948	1983	25606	13.14	12.94	333045	329908
FR20	36	1245	176	4.89	3.28	193236	182808
FR21	262	1543	2284	8.72	6.62	226205	206624
FR22	185	1707	1246	6.74	3.83	143938	124185
FR23	47	1234	245	5.21	3.03	143586	121474
FR24	377	2283	3525	9.35	7.44	160294	157123
FR25	195	2338	3718	19.07	17.66	26576	24975
FR26	547	449	4116	7.52	15.87	59709	61324
FR27	163	353	4299	26.37	27.53	145995	148922
FR28	327	2788	4781	14.62	9.76	134048	130910
FR29	102	2031	609	5.97	4.21	22520	20549
FR30	40	783	236	5.90	4.28	139181	125542
FR31	196	1597	1804	9.20	6.04	188855	165505
FR32	463	2588	5229	11.29	8.03	50505	51413
FR33	433	2728	6004	13.87	9.07	69377	63078
FR34	286	2088	5857	20.48	13.36	38141	37197
FRc0	3646	0	540241	171.72	99.90	12193161	12193161
FRc1	1062	2584	173797	163.65	91.23	2229003	2265247
FRc2	523	3123	58366	111.60	114.44	3892543	3922481
FRc3	226	3420	33041	146.20	70.56	548048	558086
FRc4	160	3486	25044	156.52	75.47	320432	323169
FRc5	55	3591	7847	142.67	71.64	61761	60285
FRc6	869	2777	131174	150.95	96.62	1995302	1983097
FRc7	2088	1558	351073	168.14	94.18	4459338	4523902
FRc8	100	3546	20246	202.46	161.41	307744	316592
FRc9	600	3046	113905	189.84	103.57	1078183	1095993
FRc10	302	3344	26627	88.17	77.64	1306425	1274670
FRc11	906	2740	142619	157.42	100.21	2324444	2358580
FRc12	1500	2146	250676	167.12	99.00	3224586	3284517
FRc13	32	3614	6653	207.91	145.33	11959	10634
FRc14	506	3140	75603	149.41	85.63	1311912	1331984
FRd0	94	0	540250	5747.35	1957.11	3548178	3548178
IT1	377	0	24090	63.90	61.89	225351	225351
IT2	395	201	24157	61.16	77.51	409889	408692
IT3	1002	2020	54918	54.81	71.37	1235378	1193338
IT4	201	507	14964	74.45	82.42	246609	248562
IT5	204	1005	10567	51.80	55.68	279014	272310
IT6	51	506	5582	109.45	101.52	57446	51211
IT7	2000	4001	98693	49.35	60.97	2849914	2812238
IT8	186	1023	2412	12.97	15.25	316602	286285
IT9	1510	4004	71167	47.13	58.08	1703944	1702002
IT10	705	3008	26809	38.03	41.62	401998	403307
ITp0	99	0	277220	2800.20	1619.86	1567576	1567576
ITp1	50	49	131773	2635.45	1401.23	742229	727038
ITp2	30	69	93666	3122.21	1599.56	266696	272316
ITp3	20	79	45854	2292.72	1128.38	264824	259988
USA0	3108	0	8070785	2596.78	3437.29	34077841	34077841
USA1	1015	2093	1876151	1848.42	916.86	5855813	5902784
USA2	103	3005	101411	984.57	341.47	527136	535608
USA3	54	3054	306284	5671.93	4488.99	604043	597371
USA4	2011	1097	4169235	2073.21	1786.40	14767588	14926726
USA5	202	2906	404093	2000.46	1994.32	8789633	8893748
USA6	504	2604	949238	1883.41	1041.57	2125887	2155981
USA7	806	2302	4234740	5254.02	5626.18	5003104	5099317
USA8	352	2756	2723212	7736.40	7741.02	4147054	4234376
USA9	1507	1601	2877429	1909.38	1517.28	10099598	10234438
USA10	13	3095	14123	1086.37	343.73	58212	53513
USA11	32	3076	205989	6437.17	4105.95	22496	24085
USA12	1004	2104	1292835	1287.68	563.79	9704950	9735646
USA13	207	2901	207785	1003.79	352.24	1307774	1326018
USA14	301	2807	312955	1039.72	394.71	2054878	2085408

Figure 2. **Normalized Mean Absolute Error (a) and Normalized Root Mean Square Error (b) for the 80 case-studies. The red squares represent the errors obtained with the value of β optimised from data on the case-study network. Black plain triangles represent the average errors obtained with β values estimated with the rule linking β and the average surface of the units obtain with the cross-validation; Dark bars represent the minimum and the maximum errors obtained with the estimated β but in most cases they are too close to the average to be seen. The green circles represent the errors obtained with the random model. The green circles represent the errors obtained with the random model. The blue triangles represent the value obtained with the radiation model. The purple cross represent the errors obtained with the modified version of the radiation model.**

Figure 3. **Comparing the predictions of the radiation model with ours for the case study $FR2$.**

Figure 4. **Comparing the predictions of the radiation model with ours for the case study $FR3$.**

Figure 5. **Comparing the predictions of the radiation model with ours for the case study $FR4$.**

Figure 6. **Comparing the predictions of the radiation model with ours for the case study $FR5$.**

Figure 7. **Comparing the predictions of the radiation model with ours for the case study $USA1$.**

Figure 8. **Comparing the predictions of the radiation model with ours for the case study $USA2$.**

Figure 9. **Comparing the predictions of the radiation model with ours for the case study $USA3$.**

Figure 10. **Comparing the predictions of the radiation model with ours for the case study $USA4$.**
